# Positive and Negative Effects of Habitat-Forming Algae on Survival, Growth and Intra-Specific Competition of Limpets

**DOI:** 10.1371/journal.pone.0051601

**Published:** 2012-12-10

**Authors:** Ezequiel M. Marzinelli, Michael T. Burrows, Angus C. Jackson, Mariana Mayer-Pinto

**Affiliations:** 1 Centre for Research on Ecological Impacts of Coastal Cities, School of Biological Sciences, University of Sydney, Sydney, New South Wales, Australia; 2 Ecology Department, Scottish Association for Marine Science, Scottish Marine Institute, Oban, Argyll, United Kingdom; 3 Environmental Research Institute, North Highland College, University of the Highlands and Islands, Thurso, Caithness, United Kingdom; University College Dublin, Ireland

## Abstract

Understanding the effects of environmental change on the distribution and abundance of strongly interacting organisms, such as intertidal macroalgae and their grazers, needs a thorough knowledge of their underpinning ecological relationships. Control of grazer-plant interactions is bi-directional on northwestern European coasts: grazing by limpets structures populations of macroalgae, while macroalgae provide habitat and food for limpets. Scottish shores dominated by the macroalga *Fucus vesiculosus* support lower densities and larger sizes of limpets *Patella vulgata* than shores with less *Fucus*. These patterns may be due to differences in inter-size-class competitive interactions of limpets among shores with different covers of *Fucus*. To examine this model, densities of small and large limpets were manipulated in plots with and without *Fucus*. Amounts of biofilm were measured in each plot. The presence of *Fucus* increased survival but hindered growth of small (15 mm TL) limpets, which were negatively affected by the presence of large limpets (31 mm TL). In contrast, large limpets were not affected by the presence of *Fucus* or of small limpets. This suggests the occurrence of asymmetric inter-size-class competition, which was influenced by the presence of macroalgae. Macroalgae and increased densities of limpets did not influence amounts of biofilm. Our findings highlight the role of interactions among organisms in generating ecological responses to environmental change.

## Introduction

Macroalgae play an important role in marine systems. Seaweeds play a critical role in primary production and are very effective sinks of carbon [1]–[3]. In addition, they provide habitat and food for many organisms [4], [5]. Environmental changes are affecting their distribution and abundances, which, in turn, affects the structure of assemblages of organisms that use them as a resource directly and indirectly, by altering ecological interactions among these organisms [6].

Limpets feed on microbial films or biofilms, which are primarily composed of microalgae, diatoms and spores of adult macroalgae [5], although macroalgae may be prevalent in diets where available [7]. Grazing of sporelings by limpets may thus influence the structure of populations of macroalgae [5], [8]–[10]. Adult macroalgae, on the other hand, provide habitat to limpets, with juveniles often aggregating under patches of plants [11]. Percentage cover of macroalgae influences distribution and abundance of intertidal gastropods among areas on the shore [12]. Also, macroalgal canopies can influence covers of microbial films, which vary at small spatial scales [13], [14]. Macroalgae can thus influence food for limpets directly by their availability for consumption and indirectly by changing the biofilm. Finally, microbial food resources are likely to be directly affected by climatic extremes because their abundances are influenced by the weather, being usually scarce in summer [14]–[16]. Such complex interactions are rarely taken into account in predicting effects of disturbances on organisms and this study should bring greater predictive ability and a better basis for management and conservation of rocky intertidal areas [6].

Competition for resources amongst organisms inhabiting intertidal rocky shores has been extensively studied [17]–[21]. Gastropods compete for microbial and macroalgal food among different species (inter-specific competition; e.g. [19]) and among individuals of the same species (intra-specific competition; e.g. [22]). Intra-specific competition is likely to be stronger, because individuals of the same species share more similar resources [23], [24]. Survival and growth of the limpet *Patella vulgata*, for example, are less affected by inter-specific than by intra-specific competition [18]. Along with other interactions between grazers and algae, such competitive interactions shape the structure of intertidal assemblages.

**Figure 1:**
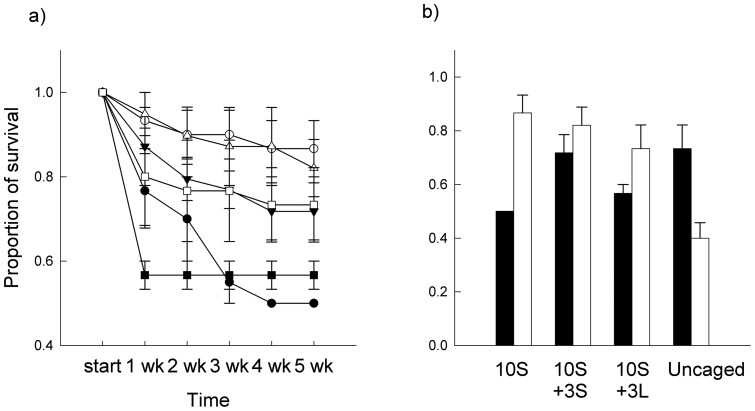
Survival of small limpets. Mean (± SE; *n* = 3) proportion of survival of small *P. vulgata* for each week (wk; a) or at the end of the experiment (b). Treatments are: 10S (circles), 10S +3S (triangles), 10S +3L (squares). S, small limpets; L, large limpets. Black symbols and bars, without macroalgae; white symbols and bars, with macroalgae.

**Table 1:** Analyses of proportion of survival of a) small or b) large limpets.*

	a) Small			b) Large		
	C = 0.35^ns^			C = 0.54^ns^		
Source	*df*	MS	*F*	*df*	MS	*F*
Ma	1	0.2022	17.94 **	1	0.0094	0.82^ ns^
Tr	2	0.0227	2.01^ ns^	2	0.0432	3.78^ ns^
Ma x Tr	2	0.0285	2.53 ^ns^	2	0.0051	0.44 ^ns^
Residual	12	0.0113		12	0.0114	

* Macroalgae (Ma) was fixed with 2 levels (+/−); Treatment (Tr) was fixed and orthogonal with 3 levels. The replicates were the plots (*n* = 3). Cochran's test (C) was used to test assumptions of homogeneity. *, P<0.05; ns, P>0.05.

*Patella vulgata* is one of the most common and abundant species of limpets in the NE Atlantic, ranging from northern Norway to southern Portugal [25], [26]. This limpet is a generalist grazer and has been shown to preferentially aggregate beneath the macroalgae *Fucus vesiculosus* [12], [27]. Evidence suggests, however, that this species is being replaced toward the southern end of its geographical range by *Patella depressa* in response to increases in temperature [25], [28]. Understanding the interactions occurring on the shores among *P. vulgata*, macroalgae and biofilm is, therefore, important to predict the effects that climatic changes will have on these systems.

**Figure 2:**
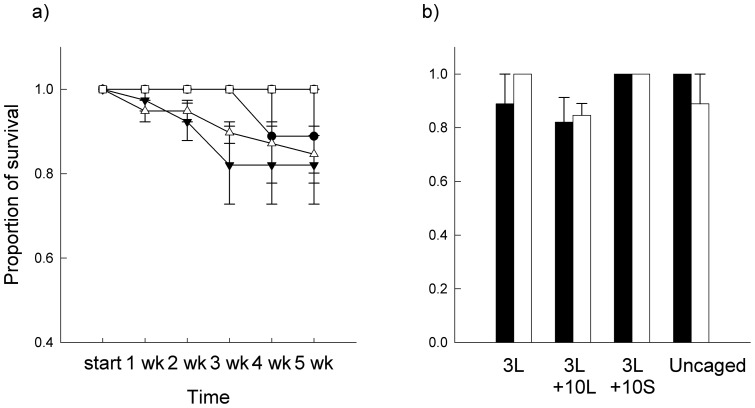
Survival of large limpets. Mean (± SE; *n* = 3) proportion of survival of large *P. vulgata* for each week (wk; a) or at the end of the experiment (b). Treatments are: 3L (circles), 3L +10L (triangles), 3L +10S (squares). S, small limpets; L, large limpets. Black symbols and bars, without macroalgae; white symbols and bars, with macroalgae.

Although there are several studies of interactions between macroalgae and grazers (see references above), these rarely consider the influence of amount and composition of biofilm (but see [13], [29], [30]). Biofilms of epilithic microphytobenthos (hereafter biofilm) have been difficult to reliably assess and manipulate *in situ* without destructive methods, but recent advances have allowed non-destructive field-based quantification of chlorophyll-*a* (as a proxy for biomass of biofilm; e.g. [31]). The approach uses remote sensing with digital cameras, enabling repeated observations to be made of the same area without interfering with any experiment. Large areas can be sampled in a single image and data can be sampled at any spatial scale within the image. Very fine resolutions are possible (<1 mm), which allow collection of data at relevant scales not possible with conventional methods.

**Figure 3:**
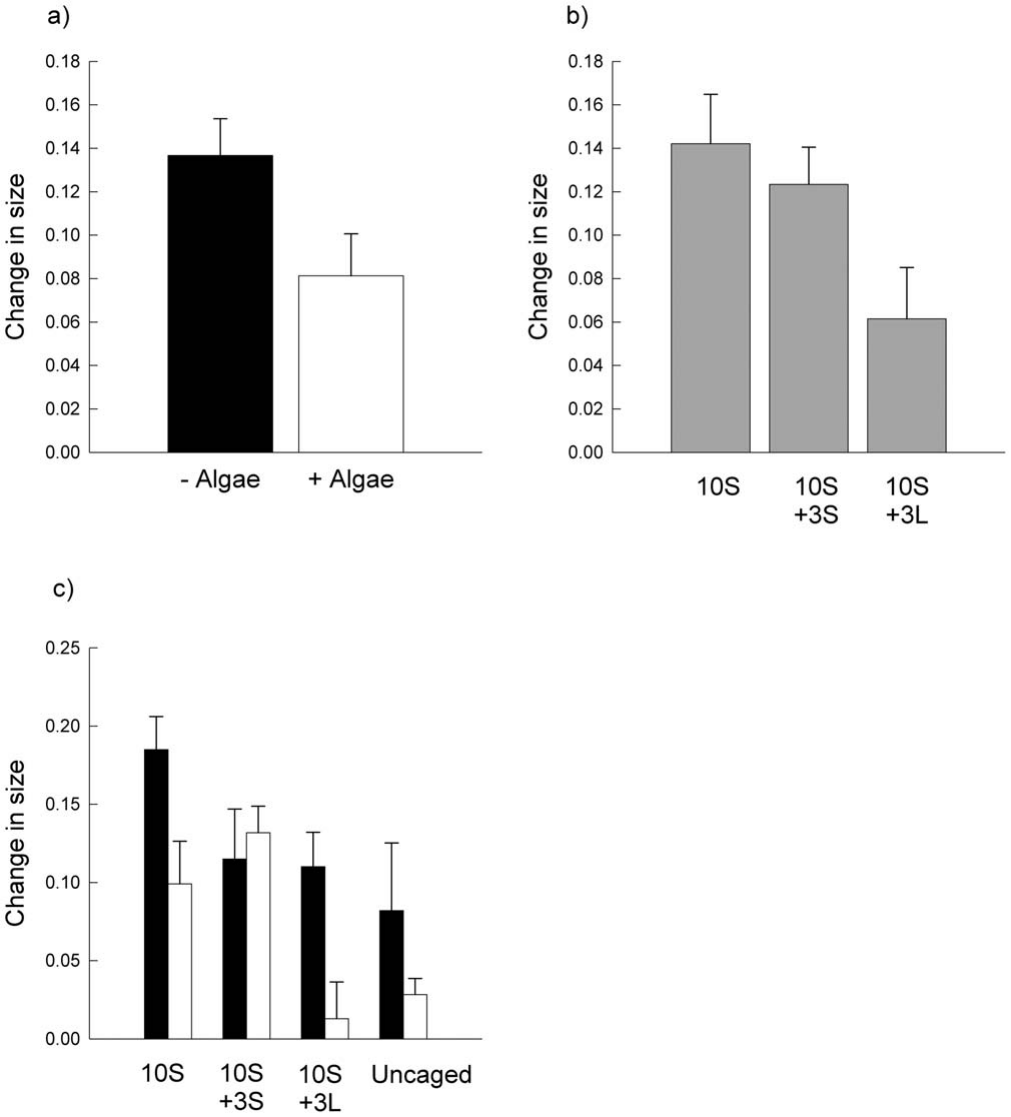
Changes in size of small limpets. Mean (± SE) changes in size of small *P. vulgata* in absence (black bars) or presence (white bars) of macroalgae (*n* = 12; a), for each treatment (*n* = 8; b) or for both (*n* = 4; c) at the end of the experiment. S, small limpets; L, large limpets.

**Table 2:** Analyses of changes in size, shell-weight and body-weight of small limpets.*

		Size		Shell-weight		Body-weight	
		C = 0.33^ ns^		C = 0.43^ ns^		C = 0.39^ ns^	
Source	*df*	MS	*F*	MS	*F*	MS	*F*
Ma	1	0.018	7.67 *	0.001	0.02^ns^	0.018	0.24^ ns^
Tr	2	0.014	5.92 *	0.038	2.42^ns^	0.251	3.41^ ns^
Ma x Tr	2	0.008	3.29 ^ns^	0.073	4.65 *	0.095	1.29^ ns^
^x^Pl (Ma x Tr)	6	0.001		0.019		0.069	
Residual	12	0.003		0.014		0.076	

* Macroalgae (Ma) was fixed with 2 levels (+/−); Treatment (Tr) was fixed and orthogonal with 3 levels; Plots (Pl) was nested in Ma x Tr with 2 levels. The replicates were the limpets (*n* = 2). Cochran's test (C) was used to test assumptions of homogeneity. Non-significant interactions (^x^) were pooled when ns with *P*<0.25. *, *P*<0.05; ns, *P*>0.05.

Preliminary observations showed a negative correlation between sizes of *P. vulgata* and their densities and between densities of small and large limpets. In particular, densities of limpets are smaller and their sizes are greater on shores with greater cover of macroalgae than on those with less cover [32]. Observed patterns of distribution and abundances of *P. vulgata* may be due to size-structured competition, which in turn may depend on the availability of biofilm or macroalgae as food. This paper therefore aimed at investigating the effect of macroalgae on competitive interactions of *P. vulgata*. If the observed differences in sizes and densities of small (12–17 mm) and large (29–34 mm) limpets are due to inter-size-class competition, it was predicted that survival and growth of small and large limpets will (i) decrease with increasing densities and (ii) be smaller in plots to which large limpets had been added than in plots to which small limpets had been added. In addition, it was predicted that survival and growth of large and small limpets will be greater in plots with macroalgae than in those without macroalgae (iii). Amounts of biofilm were predicted to (iv) decrease with increasing densities of limpets, (v) be smaller in plots with large limpets and (vi) in plots without macroalgae.

**Figure 4:**
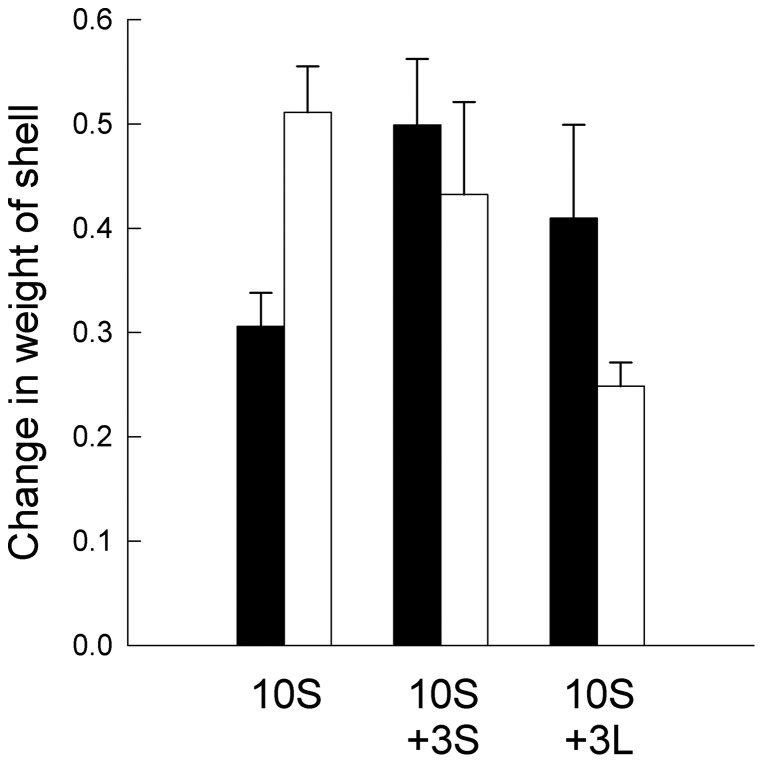
Changes in shell-weight of small limpets. Mean changes in shell-weight (± SE; *n* = 4) of small *P. vulgata* for each treatment at the end of the experiment. S, small limpets; L, large limpets. Black bars, without macroalgae; white bars, with macroalgae.

## Methods

### Inter- and intra-size class competition

Competitive interactions between size-classes of *P. vulgata* in presence or absence of macroalgae were studied at Dunbeg (56°27′N, 5°26′W), Oban, on the west coast of Scotland. Experiments were done in the mid-intertidal (approx. 2.4 m above MLW), among an area with 85± SE 6% cover of the macroalgae *Fucus vesiculosus* (hereafter *Fucus*) on a sheltered shore, with 3± SE 3% cover of *Fucus serratus* and occasional patches of red algae *Palmaria palmata* (2± SE 1%). At this location, natural densities of large (29–34 mm) and small (12–17 mm) limpets were of approximately 3 and 10 individuals per 625 cm^2^ respectively.

**Table 3:** Analyses of changes in size, shell-weight and body-weight of large limpets.*

		Size		Shell-weight		Body-weight	
		C = 0.70 **		C = 0.74 *		C = 0.25^ ns^	
Source	*df*	MS	*F*	MS	*F*	MS	*F*
Tr	4	0.0002	0.08^ ns^	0.0034	0.75^ ns^	0.0938	1.43^ ns^
Pl (Tr)	5	0.0027	3.16^ ns^	0.0046	2.06^ ns^	0.0654	5.11*
Residual	10	0.0009		0.0022		0.0128	

* Treatment (Tr) was fixed with 5 levels (3L, + macroalgae; 3L +10S, +/− macroalgae; 13L, +/− macroalgae); Plots (Pl) was nested in Tr with 2 levels. The replicates were the limpets (*n* = 2). Only 1 tagged individual remained from the 3L treatment without macroalgae. So, this treatment was not included in the analysis and the factor Macroalgae was removed. Cochran's test (C) was used to test assumptions of homogeneity. Non-significant interactions (^x^) were pooled when ns with *P*<0.25. *, *P*<0.05; ns, *P*>0.05.

**Table 4:** Summary of the effects of macroalgae (Ma) and competition (Co) on small (S, 15 mm) and large (L, 30 mm) limpets.*

			*Effect*		
*Response*		Size	Macroalgae	Competition	Ma x Co
Survival		S	−Ma<+Ma **	ns	ns
		L	ns	ns	ns
Growth	Size (TL, mm)	S	−Ma>+Ma *	10S, 13S>10S+3L *	ns
	Shell-weight (g)	S			+Ma: 10S, 13S>10S+3L −Ma: 10S = 13S = 10S+3L
	Body weight (g)	S	ns	ns	ns
	Size (TL, mm)	L	ns	ns	ns
	Shell-weight (g)	L	ns	ns	ns
	Body weight (g)	L	ns	ns	ns

* TL, total length. **, *P*<0.01; *, *P*<0.05; ns, *P*>0.05.

Experimental manipulations of densities of small and large limpets were done in plots with or without *Fucus* to determine the effects of macroalgae on competitive interactions. Three treatments were used to study the effects of large limpets (L) on small limpets (S): *i*) 10S; *ii*) 10S +3L; *iii*) 10S +3S. This allowed testing effects of adding large limpets at their natural densities to small limpets and contrasting this with effects of adding small limpets at that density (inter-*vs* intra-size-class competition on small limpets). Similarly, three treatments were used to study the effects of small limpets on large limpets: *iv*) 3L; *v*) 3L +10S (this is the same as treatment *ii* above); *vi*) 3L +10L. This allowed testing effects of adding small limpets at their natural densities to large limpets and contrasting this with effects of adding large limpets at that density (i.e. inter-*vs* intra-size-class competition on large limpets). In this way, effects of small and large limpets can be identified without being confounded with different densities [33], [34].

**Figure 5:**
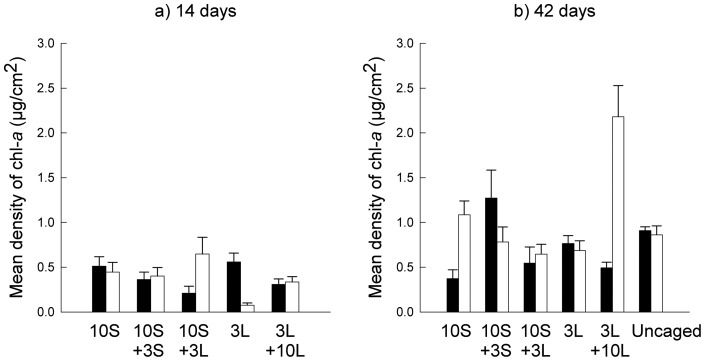
Chlorophyll-*a* densities for each treatment. Mean (± SE; *n* = 10) density of chlorophyll-*a* (mug/cm^2^) in plots with (white bars) or without (black bars) macroalgae 14 days (a) or 42 days (b) after the start of the experiment. S, small limpets; L, large limpets.

**Table 5:** Analyses of mean densities of chlorophyll-*a* for each treatment 14 or 42 days after the start of the experiment.*

		14 days			42 days	
Source	*df*	MS	*F*	*df*	MS	*F*
Ma	1	0.01	0.01 ns	1	39.1	4.43 ns
Tr	4	0.29	0.29 ns	4	8.1	0.91 ns
Ma x Tr	4	1.62	1.59 ns	4	6.0	Pooled
Pl (Ma x Tr)	20	1.02	4.28 **	10	9.9	Pooled
Residual	270	0.24		180	0.3	

* Macroalgae (Ma) was fixed with 2 levels (+/−); Treatment (Tr) was fixed and orthogonal with 5 levels; Plots (Pl) was nested in Ma x Tr with 3 levels (*n* = 10). Plots had 2 levels for analysis at 42 days because 1 plot was lost during a storm; so, 1 plot was excluded at random from all other treatments to balance the design. Cochran's test (C) was used to test assumptions of homogeneity. Non-significant interactions were pooled when ns with *P*<0.25. **, *P*<0.01; ns, *P*>0.05.

**Table 6:** Analysis of mean densities of chlorophyll-*a* for caged and uncaged treatments with limpets at natural densities (10S +3L) at the end of the experiment.*

Source	*df*	MS	*F*
Tr	1	2.50	0.73 ns
Ma	1	0.02	0.01 ns
Tr x Ma	1	3.43	17.14 ns
Pl (Tr x Ma)	8	0.16	0.05 **
Residual	108	0.20	

* Treatment (Tr) was a fixed factor with 2 levels (caged *vs* uncaged); Macroalgae (Ma) was fixed and orthogonal with 2 levels (+/−); Plots (Pl) was nested in Tr x Ma with 3 levels (*n* = 10). Cochran's test (C) was used to test assumptions of homogeneity. **, *P*<0.01; ns, *P*>0.05.

Limpets were placed in 30 roofed cages made of 4 mm plastic mesh. The basal dimensions of the cages were 27×27 cm, with a height of 30 cm. These dimensions allowed *Fucus* inside cages (1–2 individuals per cage) to move naturally when submerged and to lie naturally on the rock at low tide. The cages were attached to the rock using rapid-setting cement (Evo-Stik, Evode Industries Ltd, Dublin, Ireland). A 25×25 cm quadrat was placed on the rock and a cement strip of approximately 4 cm width and 3 cm height was formed around it, enclosing 625 cm^2^ of substratum. Cages were placed on the setting cement. A plastic straw was used to form a hole (5 mm diameter) through the setting cement to prevent accumulation of water during ebb tides and/or rainy periods. All limpets were removed from all cages using putty knives; macroalgae were removed from half of the cages using knives. The mean percentage cover of encrusting organisms inside plots was 28.9± SE 2.3. The cages were left for a week before experimental limpets were placed to allow the remnants of cement to be washed off. Each of the 5 treatments above (*i*, *ii*, *iii*, *iv*, *vi*) was randomly assigned to 3 cages with and 3 cages without macroalgae.

Large (31.3± SE 0.2 mm) and small (15.8± SE 0.2 mm) limpets were collected from adjacent areas on the shore using putty knives and taken to the laboratory. Because large limpets were smaller than the maximal size [35], there was still scope for growth, allowing testing hypotheses about growth for both size classes. Limpets were marked with nail-polish (different colours for L or S) and their maximum shell-length was measured. All large and half of the small limpets were tagged individually and weighed. All limpets were then placed underwater in 10 l plastic tanks with running sea-water for approximately 15 hours. Tagged limpets were subsequently weighed underwater (immersed weight) as described in Palmer [36], [37] to estimate shell and body mass in a non-destructive way. Because only half of the small limpets were tagged individually to follow their growth, the number of replicates for analyses of changes in size and weight throughout the experiment was smaller than for survival, which included limpets tagged individually and those marked with nail-polish at the start of the experiment. Limpets were taken back to the shore and randomly assigned to the treatments/cages. Cages were checked daily for the first 5 days (11–15 May 2009), during which dead limpets were replaced as described above (a standard procedure for these types of manipulative experiments to increase the number of animals that survived the transplantation; [33], [38]). The mortality of large and small limpets during the 5 days before the experiment started was 33% and 55% respectively. Survival was recorded weekly during the rest of the experiment. Dead animals were replaced with individuals of the same size-class collected from adjacent areas on the shore to maintain densities throughout the experiment. These replacements were marked *in situ* with nail-polish of a different colour to distinguish them from the original experimental limpets. Limpets were re-marked as required. Growth and survival rates calculated thus apply only to limpets introduced at the start of the experiment (15 May 2009).

After 45 days (25 June 2009), original limpets were removed from each cage, weighed (see above) and their maximum shell-length was measured. The measured immersed weight corresponds to the mass of the shell because the density of the body-tissue is similar to that of sea-water. The mass of the shell was estimated using a regression of dry shell weight (from dissected individuals; *n* = 20) on immersed shell weight (y = 1.602 x +0.009; R^2^ = 0.99). The mass of body-tissue was then estimated by subtraction of the estimated mass of shell from the weight of the individual in air [36].

The maximum length of frond and wet weight of *Fucus* in cages with macroalgae were measured *in situ* at the end of the experiment.

### Controls

To determine any potential effects of confinement, growth and survival of caged limpets at their natural densities (*ii*, 3L +10S) were compared with those of undisturbed limpets: 3L +10S in 6 unfenced plots of 25×25 cm. Macroalgae were removed from half of the unfenced plots. The area of removal of macroalgae was of 1 m^2^ to prevent fronds of adjacent macroalgae to enter the 25×25 cm plots. Limpets were marked *in situ* with nail-polish.

To control for any potential artefacts due to the removal and transplantation of limpets, 3L +10S limpets were disturbed in the same manner as required by the transplantation, but were placed back in unfenced plots (n = 3 plots with or without macroalgae). Finally, to control for potential artefacts due to the roof of the cages (e.g. increased shading, altered water-flow, etc.), 3L +10S limpets were disturbed as required by the transplantation, but were placed in fenced plots without roofs (n = 3 plots with or without macroalgae). Fences of 5 cm height made of plastic mesh were cemented to the rock (as with cages; see above), enclosing 625 cm^2^ of substratum. Remarkably, over 90% of disturbed limpets in plots without roofs, either fenced or unfenced, disappeared after a day. In fenced plots, many tagged empty shells were found, suggesting strong predation (possibly by crabs; see [39]). These control treatments were set again, with the same result. Data from these treatments were therefore not included.

### Estimation of biofilms

To test the hypothesis of differences in biofilm, amounts of chlorophyll-*a* were compared among treatments at the middle (28 May, day 14) and at the end of the experiment (24 June, day 42). Separate series of near infra-red (NIR) and colour images were acquired for each plot using a Fuji IS1 camera with an IR pass filter (Hoya R72) or UV-NIR cut filter (B&W 486). In plots with macroalgae, these were manually displaced to uncover most of the rock so that images could be taken. Data were acquired under natural sunlight in clear-sky conditions between 10 am and 3 pm. For all images, the user set the required aperture and the camera automatically set the appropriate shutter speed. For consistency, the white reference was set to a standard setting (“Sunny day”). Exposure-bracketing was used to provide a range of shutter speeds so an image of appropriate exposure could be chosen. Images in JPEG format are 3696×2464 pixels, collected from a height of approximately 1 m, thus each pixel in the image measuring an area of ∼0.35×0.35 mm.

Images of the plots were also used to estimate the area of available substratum (bare rock) to avoid possible confounding effects. There were no differences in the area of available substratum among treatments (ANOVA; Macroalgae x Treatment: *F*_4,20_ = 0.48, *p*≥0.75; Macroalgae: *F*_1,20_ = 1.46, *p*≥0.24; Treatment: *F*_4,20_ = 1.24, *p*≥0.33).

### Calibration

To compensate for differences in solar illumination (cloud, haze, direct sunlight) and complex indirect reflections from surrounding structures or environment, which cause variable brightness among images, images were calibrated to reflectance on a band-by-band basis. This allows amounts of chl-*a* to be compared among places and times. We used the effective 2-stage method of calibration described in detail by Murphy et al. [40] with two different reflectance standards of different brightness (respectively, 20% and 50% reflective) made from spectrally-flat material (Zenith Alucore, Sphereoptics).

The Ratio Vegetation Index (RVI) was selected to quantify amounts of chlorophyll from the images because it is simple to implement and has been used in several previous studies to quantify amounts of chlorophyll in intertidal areas [31], [41]. This index is calculated from calibrated image data using the equation: RVI  =  NIR/red. Values of RVI were calculated for ten regions of interest (ROI; each 0.4×0.4 cm) placed in each plot on areas of apparently bare substratum, i.e. avoiding barnacles or foliose or encrusting macroalgae or lichen.

### Laboratory vs camera estimates of chlorophyll

To determine the relationship between chlorophyll and reflectance 30 sandstone cores with a range of amounts of biofilm (estimated visually) were collected from the area of shore used in the experiment. Cores were rinsed in clean sea-water and then photographed. The surface area of the upper surface of the sandstone core on which biofilm could grow was calculated precisely using standard image analysis. Chlorophyll was extracted from the cores using the cold methanol method [42]. Chlorophyll concentration was determined by spectrophotometry and amounts standardized to the surface area of the rock core [42] and expressed per unit area in µg.cm^−2^ [43].

Vegetation indices of the upper-surface of the sandstone cores were calculated by extracting the pixel values over each core and averaging them. This was done separately for red and NIR reflectance. These averages were then used to calculate RVI for each core. Laboratory and image estimates of chlorophyll (RVI) were compared using linear regression. This regression equation was then used to calculate mean amounts of chlorophyll-*a* for each experimental plot from the respective values of RVI.

### Analysis of data

Analyses of variance (ANOVA) were used to examine differences among means. Cochran's test (*C*) was used to test assumptions of homogeneity. When Cochran's test for heterogeneity of variances was significant and no transformation was possible, the analysis of variance was still done because it is robust to departures from the assumptions when sample size is large [44]. Where significant interaction terms were detected, Student-Newman-Keuls (SNK) comparisons of means were used to determine which treatments differed [44]. All tests were done using GMAV 5 statistical software for Windows [45].

## Results

### Survival

Survival of small limpets was greater in plots with macroalgae (81%) than in plots without macroalgae (59%; Table 1a, Fig. 1a), such that macroalgae reduced mortality by a factor of two. There were no significant differences among density treatments nor interactions of density and presence of macroalgae. Survival in plots with macroalgae to which small limpets were added (10S +3S) was greater than in those with large limpets (10S +3L; Fig. 1a,b). Survival of small limpets in uncaged control plots without macroalgae did not differ from that of caged limpets at natural densities. In contrast, survival of small limpets in uncaged control plots with macroalgae was significantly smaller than that of caged limpets at natural densities (Fig. 1b).

There was no effect of macroalgae on the survival of large limpets (Table 1b). Although survival of large limpets did not differ significantly among treatments, it was smallest in plots to which large limpets were added (3L +10L; Fig. 2a,b). Survival of caged or uncaged large limpets at natural densities did not differ, independently of the presence or absence of macroalgae (Fig. 2b).

### Growth

At the start of the experiment, mean sizes of small (15.8± SE 0.2 mm) or large (31.3± SE 0.2 mm) limpets did not differ among treatments (ANOVA; small, *F*_5, 12_ = 1.54, *P*≥0.3; large, *F*_5, 12_ = 0.84, *P*≥0.6). After 5 weeks, changes in size (i.e. [size _end_ – size _start_]/size _start_) of small limpets in plots without macroalgae were greater than in plots with macroalgae. In addition, changes in size of small limpets in 10S and 13S treatments were significantly greater than those under the 10S +3L treatment (SNK tests; Table 2, Fig. 3a,b). This difference in sizes appeared to be greater in presence of macroalgae (Fig. 3c), although there were no significant Algae x Treatment interactions (Table 2). Changes in size of caged small limpets did not differ from those in uncaged plots at natural densities (10S +3L; ANOVA; *F*_1, 8_ = 0.07, *P*≥0.8; Fig. 3c).

Analysis of changes in shell-weight of small limpets showed a significant Algae x Treatment interaction (Table 2). In presence of macroalgae, changes in shell-weight of small limpets under the 10S and 13S treatments were greater than those under the 10S +3L treatment (Fig. 4). SNK tests did not show, however, where the differences were. In contrast, changes in body-weight of small limpets did not differ between plots with or without macroalgae or among treatments (Table 2).

Finally, there were no differences in changes in size, shell-weight or body-weight of large limpets among treatments, independently of the presence or absence of macroalgae (Table 3). Changes in size of caged large limpets did not differ from those in uncaged plots at natural densities (10S +3L; ANOVA; *F*_1, 8_ = 0.06, *P*≥0.9). Results are summarized in Table 4.

### Macroalgae and biofilm

At the end of the experiment, there were no differences in the maximum length of frond (27.2± SE 1.6 cm) or wet weight (242± SE 18 g) of *Fucus* among treatments with macroalgae (ANOVA; length, *F*_5, 12_ = 0.7, *P*≥0.6; wet weight, *F*_5, 12_ = 0.8, *P*≥0.6).

Densities of chlorophyll-*a* did not differ among treatments after 14 or 42 days (end of experiment), despite great variability among plots in the former (Table 5, Fig. 5). Densities of chl-*a* in caged plots with limpets at natural densities (10S +3L) did not differ from those in uncaged plots at natural densities (Table 6, Fig. 5b).

## Discussion

Survival of small limpets was greater in the presence of *Fucus*, with mortality reduced from 41% to 19%. This was expected since macroalgae have been shown to ameliorate environmental conditions [12], [21], providing habitat and food for limpets [5], [9], [12]. There was, however, no effect of the presence of *Fucus* on the survival of large limpets. Moore et al. [12] found that the removal of *Fucus* caused increased mortality and/or relocation of home scars on individuals of *Patella vulgata* using animals over a natural range of sizes (Moore personal communication).

Competition for resources often reduce the survival of inferior competitors, particularly juveniles [33], [46]. This effect can be direct by interference competition. Some limpets actively dislodge and thereby kill inferior competitors [47]. Alternatively, the effect of competition on survival can be indirect, by exploitation of food resources leading to increased mortality and decreased growth. If the quantity of resources in each area is equal, competition would be manifested by increases in mortality at higher densities [33]. In the present study, survival of limpets appeared not to be affected by increases in densities through the addition of individuals of any particular size-class, although there was a trend for lower survival of large limpets in treatments where other large limpets were added. A similar trend of lower survival with the addition of large limpets was observed for small limpets in the presence of macroalgae. Large limpets appear, therefore, to influence the survival of smaller ones, particularly in the presence of *Fucus*.

The outcome of competitive interactions may depend on the type of habitat in which these occur [48], [49]. In terms of growth, contrary to our prediction, the presence of macroalgae had a negative effect on the competitive ability of small limpets. This could be due to limpets being reluctant to move, or incapable of moving, over *Fucus* when searching for food, affecting therefore their feeding and, consequently, their growth. In the presence of *Fucus*, the addition of large limpets reduced significantly the shell-weight of small limpets, which could affect their capacity to defend themselves from predators [47]. These results suggest the occurrence of stronger intra-specific competition in this habitat. Growth in size of small limpets was also affected by the presence of *Fucus* and large limpets, but, interestingly, there were no interactions between these two factors. Growth and survival of large limpets, on the other hand, did not seem to be affected by the addition of small limpets. Results of this experiment suggest not only that competition was occurring at natural densities, but also provide evidence of asymmetry in the competitive interactions between the two size-classes of limpets. Body-size has been considered by many authors to be an important feature on competitive superiority, leading to asymmetric interactions [17], [50]. Large individuals are usually competitively superior and their greater requirement for food could lead to this asymmetry in competitive interactions, either through exploitation or interference competition [17], [22], [47], [50]. Our results supported this general theory and are similar to those found in an experiment done with *Patella depressa*, where the effect of large limpets on small ones was greater than the other way around [22].

Although the cages used in the experiments might have had some potential effects on survival and growth of limpets at natural densities, these, unfortunately, could not be tested due to great mortality of limpets in the procedural controls (see *Methods: Controls*). Limpets in these shores have been shown to suffer from strong predation by crabs [39], which may have been enhanced due to disturbance of the limpets in roof-less control plots. Large limpets do not seem, however, to be affected by fencing [22]. Here, survival of small limpets in uncaged control plots without macroalgae did not differ from that of caged limpets at natural densities, suggesting no experimental artefacts. In contrast, survival of small limpets in uncaged control plots with macroalgae was significantly less than that of caged limpets at natural densities, suggesting potential effects due to the manipulation and/or caging of the limpets. We suspect that this effect was due to an artefact of the greater loss of nail-polish tag marks applied in wet *in situ* conditions to limpets in uncaged control plots than those applied to limpets in caged treatments in dry laboratory conditions. Boaventura et al. [22], in a similar type of experiment, also found that the loss of small limpets was greater in the unfenced control than in the fenced control.

For competition to occur, two or more individual organisms require the use of common resource(s) that are limiting and/or in short supply, such as space or food. The main source of food for many species of limpets is biofilm. A shortage of biofilm should, therefore, cause intra- and/or inter-specific competition. It is extremely hard, however, to determine how much biofilm is considered a “limiting resource” and, consequently, what amount should lead to competition. The best way to determine whether food has actually become a limiting resource would be to manipulate the amount of biofilm, i.e. having replicated plots with different known amounts of biofilm. This is, however, extremely difficult to achieve in the field, despite all the advances in this area. In general though, it is known that abundances of biofilm are smaller in summer [15], [51], [52], when the experiment presented here was done, than during cooler seasons. On rocky shores in the UK, the abundance of chlorophyll *a* (as a proxy for biomass of biofilm) varied from 2 to 4 μg cm^–2^ in summer and was considered a limiting resource in this season [51]. In Sydney, where many studies have shown the occurrence of competition among grazers on rocky shores [19], [33], [53], the amounts of chlorophyll *a* at mid-tidal heights on the shore often range between 1 and 2 μg cm^–2^ [41]. In the present study, the amount of chlorophyll *a* measured in most of the experimental plots was between 0.5 and 1.5 μg cm^–2^. It is reasonable, therefore, to assume that food was a limiting resource.

Unlike our predictions, however, amounts of biofilm did not differ among treatments. The absence of differences in amounts of chlorophyll *a* between plots with or without macroalgae was surprising. One possible explanation is that biofilms were grazed to a minimum in all treatments. This is, however, unlikely because amounts of biofilm in all treatments increased throughout the experiment. In addition, the method for estimating amounts of chlorophyll is most reliable when calibrated for surfaces of smooth, unshaded rock. Such surfaces formed only a small percentage of the plots (and were often limpet home scars), the remainder being encrusted with barnacles, algae or lichens or shaded by the cage. Some species of limpet cannot move or feed over encrusting organisms such as barnacles [46]. Given the densities of limpets and covers of encrusting organisms on the shores used in the present study, it is unlikely that this is the case for *P. vulgata*. There is also evidence that in some situations, *P. vulgata* also feed directly on *Fucus* with fragments of leathery macroalgae often prevalent in gut contents [7]. Sampling from the small proportion of the area that was ‘bare’ rock may not have been representative of the actual amounts of biofilm available as food to grazing limpets. Were it possible to quantify reliably amounts of chlorophyll *a* on the highly rugose surface of barnacles or on macroalgae, differences in amounts of microbial food among treatments may become apparent. Finally, the method used in the present study quantified standing stock of biofilm, not productivity. It is possible that productivity of biofilms did differ with presence/absence of *Fucus* and this may explain differences in growth. The lack of differences in standing stocks among the different treatments of limpets for either condition suggests that this is unlikely, but do not rule it out.

The findings of this experiment are a valuable contribution to our ability to predict possible effects of environmental changes on the ecology of rocky shores. Processes such as competition can vary from habitat to habitat (see references above). The potential mechanisms for disturbances (natural and/or anthropogenic) to influence this strongly interacting system of plants and grazers are many: from direct effects on survival and growth of the key species to indirect effects on the interactions themselves. Here, we have shown that macroalgae can influence the survival and competitive ability of small limpets and that change in the abundance of macroalgae is likely to affect the abundance of these grazers. Consequences of such effects for the diversity and composition of the rocky shore assemblage are not yet well understood. For instance, stochastic events that remove adult macroalgae and/or negatively affect their recruitment may affect the dynamics on these rocky shores. Diversity of understorey macroalgae and those species reliant on the ameliorating effects of the canopy on desiccation and heat stress may decline, while those organisms that may compete for space with macroalgae may become more diverse. Studies of interactions that manipulate and assess as many trophic levels involved as possible will make for much better predictions about the impacts of disturbances and environmental change on complex ecological systems.
